# Cost-effectiveness of live oral attenuated human rotavirus vaccine in Tanzania

**DOI:** 10.1186/s12962-015-0033-0

**Published:** 2015-04-28

**Authors:** George M Ruhago, Frida N Ngalesoni, Bjarne Robberstad, Ole F Norheim

**Affiliations:** School of Public Health and Social Sciences, Muhimbili University, P.O Box 65015, Dar es Salaam, Tanzania; Ministry of Health and Social Welfare, P.O Box 9083, Dar es Salaam, Tanzania; Department of Global Public Health and Primary Care, University of Bergen, Bergen, Norway; Centre for International Health, University of Bergen, Bergen, Norway

**Keywords:** Cost, Cost-effectiveness, Rotavirus, Vaccine

## Abstract

**Background:**

Globally, diarrhoea is the second leading cause of morbidity and mortality, responsible for the annual loss of about 10% of the total global childhood disease burden. In Tanzania, Rotavirus infection is the major cause of severe diarrhoea and diarrhoeal mortality in children under five years. Immunisation can reduce the burden, and Tanzania added rotavirus vaccine to its national immunisation programme in January 2013. This study explores the cost effectiveness of introducing rotavirus vaccine within the Tanzania Expanded Programme on Immunisation (EPI).

**Methods:**

We quantified all health system implementation costs, including programme costs, to calculate the cost effectiveness of adding rotavirus immunisation to EPI and the existing provision of diarrhoea treatment (oral rehydration salts and intravenous fluids) to children. We used ingredients and step down costing methods. Cost and coverage data were collected in 2012 at one urban and one rural district hospital and a health centre in Tanzania. We used Disability Adjusted Life Years (DALYs) as the outcome measure and estimated incremental costs and health outcomes using a Markov transition model with weekly cycles up to a five-year time horizon.

**Results:**

The average unit cost per vaccine dose at 93% coverage is US$ 8.4, with marked difference between the urban facility US$ 5.2; and the rural facility US$ 9.8. RV1 vaccine added to current diarrhoea treatment is highly cost effective compared to diarrhoea treatment given alone, with incremental cost effectiveness ratio of US$ 112 per DALY averted, varying from US$ 80–218 in sensitivity analysis. The intervention approaches a 100% probability of being cost effective at a much lower level of willingness-to-pay than the US$609 per capita Tanzania gross domestic product (GDP).

**Conclusions:**

The combination of rotavirus immunisation with diarrhoea treatment is likely to be cost effective when willingness to pay for health is higher than USD 112 per DALY. Universal coverage of the vaccine will accelerate progress towards achievement of the child health Millennium Development Goals.

## Background

Diarrhoea is the second leading cause of morbidity and mortality globally among children below five years of age and is responsible for 23 million Disability Adjusted Life Years (DALYs) annually, about 10% of the total global childhood disease burden [1]. The global burden of diarrhoea is highest among children in low-income countries, with countries in sub-Saharan Africa accounting for more than 50 per cent of cases worldwide [1]. In Tanzania, about fourteen per cent of all deaths in children younger than five years is due to diarrhoea, making it liable for five per cent of the total national DALYs [1,2]. About 70 per cent of the burden occurs before the first birthday. Tanzanian children under the age of five, are estimated to have 3.5 episodes of diarrhoea per year, reaching a peak frequency between 6–12 months of 4.72 episodes per year [3]. Rotavirus is the single most important cause of diarrhoea: estimated to represent about 40 per cent of all diarrhoea related morbidity and mortality in children globally [4]. Similar diarrhoea causality has been observed in a multi-country study, which included Tanzania, where 34 percent of all diarrhoea episodes were due to rotavirus [5].

The introduction of integrated management of childhood illness (IMCI) more than two decades ago strengthened the management of diarrhoea [6], with the adoption of oral rehydration solution (ORS) as a main intervention for diarrhoea treatment, recommended by World Health Organization (WHO) and United Nations Children’s Fund (UNICEF) [7]. Treatment of diarrhoea with ORS has shown marked effectiveness in preventing dehydration and reducing diarrhoea related mortality [8]. To achieve optimal effectiveness, diarrhoea treatment adopting the principles of IMCI requires large coverage and community participation. However, the recent emphasis on vertical programmes, targeting specific diseases such as Malaria, TB and HIV/AIDS, has led to reduced funding for IMCI and has weakened the management and control of diarrhoea [9].

WHO recommends including rotavirus vaccine into national immunization programmes [10]. Tanzania did this under the support of the GAVI Alliance in January 2013 [11]. Two rotavirus vaccines are currently available for Tanzania. Rotarix®, by GlaxoSmithKline, is a single strain, live attenuated human rotavirus vaccine (RV1) administered orally in two doses. RotaTeq®^,^ by Merck & Co Inc., is a live, human-bovine reassortant pentavalent rotavirus vaccine (RV5), administered orally in three doses. A third vaccine LLR, Lanzhou Institute Biomedical Products is a three dose vaccine currently licensed for use in China only, while a fourth Indian vaccine (ROTAVAC), has shown promising results but is not yet available for scale up [12]. WHO recommends that infants are vaccinated between six and fifteen weeks, and that the last dose is not given later than 32 weeks of age [13,14]. The introduction of RV1 in Tanzania offered a unique opportunity to quantify all health system implementation costs, including programme costs, during planning, piloting and scale-up of the new programme. The aim of this study was to collect primary cost data from the perspective of the health care provider and to compare the cost-effectiveness of the RV1 rotavirus vaccine to existing treatment strategies for diarrhoea in children.

## Methods

### Study setting and perspective

The study was a cost effectiveness analysis from the perspective of health service providers in Tanzania. We adopted a health provider perspective because this information would be important for national health decision makers, and because a wider societal perspective is much more data intensive and would require data that are not easily available in this setting. We compared the current treatment of diarrhoea (using oral rehydration salt (ORS) and intravenous (IV) fluid), with the addition of rotavirus vaccination to the current diarrhoea treatment and with the provision of rotavirus vaccine (RV1) alone. In addition we included a hypothetical alternative of providing no treatment to reflect further on what the outcome might be if the interventions were not implemented [15]. The pentavalent rotavirus vaccine (RV5) strategy was not included in the model analysis due to lack of cost data in Tanzania.

### Description of interventions

Treatment of diarrhoea in children with ORS and IV fluids in Tanzania follows a three-step plan (A-C) depending on diarrhoea severity, which is determined by dehydration status. Plan A should be followed for cases of mild diarrhoea, plan B for moderate and plan C for severe diarrhoea [16]. The single strain live attenuated human rotavirus vaccine (RV1) is administered to infants orally in two doses, the first dose at six weeks and the second at ten weeks [14].

### Costs

We collected primary cost data for diarrhoea management and additional costs of introducing RV1 to the national immunisation programme in two districts, purposely sampled to include a rural district (Kisarawe) and an urban district (Ilala). Costing was done from a health provider perspective. In each district we collected data from one hospital (Amana hospital for Ilala and Kisarawe hospital for Kisarawe district) and one health centre (Chanika Health Centre in Ilala and Masaki Health Centre in Kisarawe) for the one-year period July 2011 to June 2012. We collected the cost data before the introduction of rotavirus vaccine, but the preparation for the rollout was at an advanced stage, including plans for the procurement and distribution of vaccines, training of health personnel and the preparation for storage facilities. In case the available information on resource use was not sufficient we used information on other vaccines under the expanded programme on immunisation (EPI). We used a modified WHO and Joint United Nations Programme on HIV and AIDS (UNAIDS) costing tool, to identify all resource use [15,17].

#### Resource identification

We categorised health facility departments into three costing centres and applied the ingredient approach as proposed by WHO-CHOICE to identify resource use in each of the cost centres [15]. First, we identified all resources used in centres that directly provide services for child immunisation, and outpatient and inpatient departments that provide diarrhoea treatment to children. Second, we identified resources used in indirect care cost centres that provided services but not direct medical care (ancillary services). Thirdly, we included other support service cost centres such as general administrative and warehouse costs.

#### Resource measurement and valuation

Resource use was categorised into recurrent and capital goods. We classified capital items as those with useful life years above one year or costing above Tsh100000 (about 62 US$). Resource use was measured through review of available inventories such as ledgers, order books, and records of medical supplies used. All records were anonymous, only specifying resources used in treating diarrhoea or providing rotavirus vaccine. We employed a step down costing approach to allocate resources between cost centres [18]. The proportion of the number of workers at each cost centre as a percentage of total workers at the health facility was used to allocate shared resources to the cost centres. The number of diarrhoea patients among all inpatient and outpatient attendees, and the number of rotavirus doses as a percentage of all vaccine doses were used as a proxy to obtain specific resource use by each intervention.

To value all identified resources for rotavirus vaccination and diarrhoea management, we used the Tanzania Medical Stores price catalogue to assign costs for medical equipment and drugs [19]. The cost of non-medical equipment was obtained from 2011/2012 tender prices for the Government Procurement Services Agency (GPSA) [20]. Building rents were estimated as per Tanzania National Housing Corporation (NHC) rental charges obtained through interview with key personnel at NHC. All cost data were collected in Tanzania shillings (TSH) and converted to US dollars using the Bank of Tanzania Interbank average annual exchange rates for 2011 and 2012 [21].

The capital costs were annuitized using Bank of Tanzania average interest rates for 2011/ 2012 at 9.6 per cent [21], and we adopted useful life years from WHO country estimates [22]. All data were analysed using Microsoft Excel (2010).

#### Unit cost

To obtain the unit cost per immunized child, we divided the total cost by the total estimated number of children to be vaccinated with the RV1 vaccine, obtained from the current coverage levels of the existing child immunisation package (DPT- HB) from each of the study facilities. Outpatient (OPD) unit costs were obtained by dividing the total OPD cost (capital and recurrent cost) by the annual number of children with moderate diarrhoea visiting the OPD. Inpatient (IPD) unit costs were derived by dividing the total IPD cost (capital and recurrent cost) by the total number of IPD bed days specific to children admitted with severe diarrhoea. To obtain the total unit cost, the urban/rural costs were weighted using the proportion of population attending at each health facility, and the proportion of the population in each district. We assumed the constant returns to scale, i.e. the same unit prices for administration and disease management apply both with and without the intervention.

### Effectiveness

Through a systematic search we identified the most recently updated systematic reviews and meta-analyses of the effectiveness of the RV1 vaccine [14]. Only one multicentre double blinded, randomized placebo-controlled study conducted in South Africa and Malawi [23], reported rota vaccine efficacy on all-cause diarrhoea for countries with high diarrhoea mortality rates. The data analysis was conducted according to the protocol. The efficacy from this trial is used in our study. The effectiveness of diarrhoea treatment using ORS was retrieved from a systematic review by Munos et al. [24]. The effectiveness of IV fluids against severe diarrhoea were obtained from a Cochrane systematic review by Hartling et al. [25]. In our model we used vaccine efficacy against all-cause severe diarrhoea to reflect the real Tanzanian clinical settings whereby routine management of diarrhoea is based on clinical assessment criteria. Key input parameters are listed in Table 1.

**Table 1:** **Key input parameters for cost-effectiveness base case and sensitivity analyses**

**Parameter**	**Base case**	**Range**	**Distribution**	**Source**
**Cost (2012 US$)**				
Cost per fully immunised child for rota vaccine (RV1) at 93% coverage (cRotaVac)**	16.99	±25%	Gamma	Table 2
Cost per OPD visit for diarrhoea treatment (cModD)	3.84	±25%	Gamma	Table 3
Cost of in-patient diarrhoea treatment per bed day (cSevD)	8.90	±25%	Gamma	Table 4
Cost discounting rate (cDR)	0.03	0.00 – 0.06	N/A	[15]
**Disability weights**				
Disability weight moderate Diarrhoea (uModD)	0.202	0.133 - 0.299	Beta	[32]
Disability weight severe Diarrhoea (USevD)	0.281	0.184 - 0.399	Beta	[32]
Outcome discounting rate (oDR)	0.030	0.000 – 0.060	N/A	[15]
**Effectiveness (Relative Risk ratio)**				
Effectiveness of RotaVaccine on all cause diarrhoea (effRotaVac)	0.698	0.570 - 0.850	Log-normal	[23]
Effectiveness of IMCI on moderate diarrhoea (effImci_OPD)	0.590	0.430 - 0.680	Log-normal	[24]
Effectiveness of IMCI on severe diarrhoea (effImci_IPD)	0.570	0.420 - 0.660	Log-normal	[25]
**Transition Probabilities (weekly)**				
Probability of progressing from well to moderate diarrhoea (tpModD)	0.116	0.072 - 0.167	Beta	[3]
Probability of progressing from moderate to severe diarrhoea (tpSevD)	0.048	0.035 - 0.056	Beta	[27]
Probability of recurrent moderate diarrhoea (tpRecModD)	0.005	0.004 – 0.006	Beta	[28]
Probability of recurrent severe diarrhoea (tpRecSevD)	0.0038	0.003 – 0.0045	Beta	[28]
**Mortality**				
Probability of dying from diarrhoea (Case fatality rate (CFR) <5 yrs (%))	0.019	0.0119 -0.0265	Normal	[27]
(PDeath_NoInt)				
**General**				
Average number of bed days spent in hospital	4	2 - 6	N/A	Primary data
Diarrhoea treatment coverage rates	41%	44%-68%	N/A	[30]
Vaccine coverage rates ( reference to DPT-HB-Hib coverage)	93%	85% – 95%	N/A	[30]
Healthy life expectancy at birth	52	49,4 - 53,1	N/A	[32]

**In the model the vaccination cost are assigned once as transition cost to vaccinated child on first and second dose i.e. only during a monthly cycle corresponding to vaccination.

### Markov model overview

**Figure 1:**
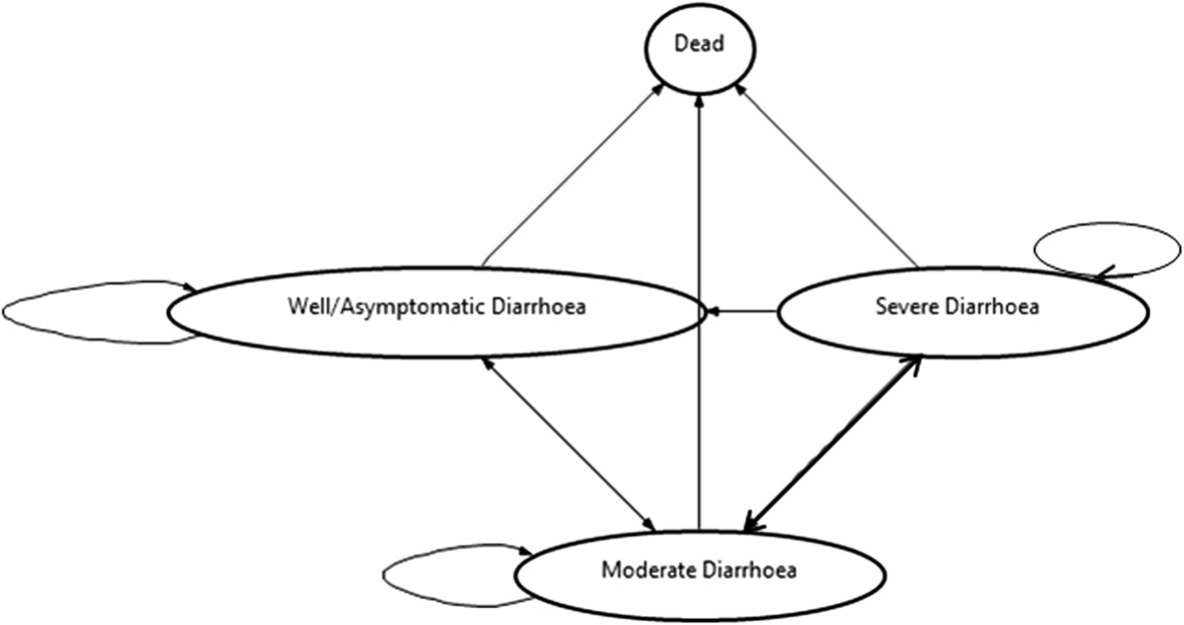
Markov model showing the health states of diarrhoeal disease, including “well/asymptomatic”, “moderate diarrhoea”, “severe diarrhoea” and “dead”, which is an absorbing state.

We constructed an individual Markov state-transition model (Figure 1) with weekly cycles with TreeAge Pro 2013 software (Williamstown, MA, USA). A five-year time horizon was adopted to reflect the fact that diarrhoea from rotavirus infection is primarily a health problem during the first five years of life [3,5,10].

For each weekly cycle in the model, children can be in one of four possible health states; well/asymptomatic infection (1), moderate diarrhoea (2), severe diarrhoea (3) and dead (4). Children in the well/asymptomatic state are exposed to diarrhoea infections. For each cycle, the child may remain well, contract moderate diarrhoea or die from other causes (background mortality). In the moderate state, children may recover from diarrhoea infection, continue with recurrent moderate diarrhoea, progress to severe diarrhoea or die from other causes. Individuals progressing to severe diarrhoea may recover, continue with recurrent moderate diarrhoea or die from either diarrhoea or other causes. The model assumptions were based on the diarrhoea classification by severity described in the Tanzania national treatment guideline [16].

### Transition probabilities

The movement between health states (as described above) is modelled on the basis of transition probabilities and the effectiveness values of the diarrhoea treatment options in the model. The probabilities were obtained from the literature. The probabilities of acquiring moderate diarrhoea infections are based on age specific incidence for Tanzania (Table 1) [3]. Yearly incidence rates were converted to weekly probabilities of diarrhoea infections using the formula p = 1- exp^(−rt)^ where p = probability, r = rate, t = time period (weekly) [26]. The transition probability of progressing from moderate to severe diarrhoea is based on a systematic review by Walker et al. [27], while the probabilities of recurrent moderate and severe diarrhoea were taken from Lamberti et al. [28] (Table 1).

To estimate the likelihood of mortality from diarrhoea infection, case fatality rates (CFR) for diarrhoea were retrieved through a literature search [27]. We used a Tanzanian life table for the year 2011 to estimate the risk of all-cause mortality, which was adjusted for diarrhoea mortality to calculate background mortality rates [29]. We assumed a reasonable target coverage of rotavirus vaccine to be equal to DPT-HB vaccine coverage (93%) [30]. We applied a dropout rate of 5% for the second dose, on the basis of the 2010 Tanzania Demographic and Health Survey (TDHS) [30].

### Health outcomes

We estimated health outcomes using disability-adjusted life years (DALYs). DALYs were calculated in the Markov model by combining years lived with disability (YLD) and years of life lost (YLL) for each weekly cycle. DALYs averted were calculated for each cycle and accumulated over the model time horizon. This was repeated for each diarrhoea management strategy [31]. DALYs averted were calculated as the difference between the treatment strategies. To obtain YLD, we used recently updated disability weights of 0.202 and 0.281 for moderate and severe diarrhoea [32]. For children in a well state a disability weight of 0 was applied, assuming all individuals in this state are either healthy or with asymptomatic diarrhoea [32]. We did not incorporate age weighting since this is not recommended in the most recent DALY guidelines [33].

To compute YLL, a disease weight of 1 reflecting the worst state (i.e., death) and a healthy life expectancy at birth for Tanzania, 52 years, was used [34]. All individuals in the state of death were assigned a weight of 1. At the final cycle all cohorts ending up in the state of wellness were assigned a final reward equal to the healthy life expectancy at two years [31].

### Cost effectiveness analysis

We used the hypothetical no intervention as a baseline and compared it to the modelled incremental cost effectiveness ratios (ICERs) of implementing the current standard of care for diarrhoea treatment in children, adding the RV1 vaccine to the current diarrhoea treatment, and RV1 vaccine given alone. The base case ICER was computed by dividing the incremental cost to incremental DALYs averted in each of the study interventions. Costs and effectiveness were discounted at an annual rate of 3% recommended by WHO for low income countries [15]. Most economic evaluation guidelines recommend discounting of both cost and effects, which is also reflected in the applied literature [18,35,36].

### Sensitivity analyses

We performed one-way sensitivity analyses to evaluate the impact of single assumptions on costs and outcomes. As upper and lower variable ranges, we used upper and lower 95% confidence limits, respectively, wherever reported in the literature. When confidence intervals were not reported and for the primary cost data we used a range of +/− 25% (Table 1). This reflects a reasonable range of variation in cost and is commonly used in cost effectiveness studies [37-39].

We used probabilistic sensitivity analysis to assess the overall robustness of the results. We did this by running the model with distributions for each parameter rather than point estimates. We computed distributions for the parameters using base case values as means, and standard errors calculated from uncertainty ranges (Table 1). For disability weights and transition probabilities, beta distributions were used since this restricts values to the range between 0 and 1. Gamma distributions were used for costs to avoid negative values [26], while, log-normal distributions were assumed for relative risks.

Monte Carlo simulation was used to draw 10,000 random samples from the distributions that were combined into cost-effectiveness pairs. The cost-effectiveness pairs were used to estimate the probability that each intervention is cost effective for a range of willingness to pay to avert DALYs. The results of the probabilistic sensitivity analysis are presented as a cost-effectiveness scatter plot and a cost-effectiveness acceptability curves.

### Research ethics

Ethical clearance was obtained from Medical Research Coordinating Committee of the National Institute for Medical Research, Tanzania. All data used in the study were anonymous; only record books without any patient identity were used. The funding agency had no influence on the study design or results.

## Results

### Costs

The total weighted average cost of rolling out RV1 vaccine at 93% coverage is US$ 8.4 per vaccine dose. The weighted unit cost per vaccine dose is US$ 5.2 in urban health facility and US$ 9.8 in rural facilities (Table 2). Recurrent costs account for 89% in urban and 87% in rural facilities. In urban facilities, 60% and, in rural facilities , 39% of the total cost is used for purchase and distribution of vaccines.

**Table 2:** **Average cost for providing rotavirus vaccine services, 2012 US$**

**Cost category**	**Urban**						**Rural**					
	**Hospital**	**%**	**Health Centre**	**%**	**Average**	**%**	**Hospital**	**%**	**Health Centre**	**%**	**Average**	**%**
**Capital Cost, N (%)**												
Buildings	582	**2.6**	517	**5.3**	550	**3.5**	504	**8.3**	265	**4.8**	384	**6.6**
Equipment	234	**1.1**	210	**2.2**	222	**1.4**	163	**2.7**	147	**2.7**	155	**2.7**
Vehicles	140	**0.6**	140	**1.4**	140	**0.9**	36	**0.6**	33	**0.6**	35	**0.6**
Training on IMCI	281	**1.3**	249	**2.6**	265	**1.7**	236	**3.9**	189	**3.4**	213	**3.7**
**Total capital costs**	**1237**	**5.6**	**1116**	**11.5**	**1177**	**7.4**	**939**	**15.4**	**634**	**11.5**	**787**	**13.5**
**Recurrent Cost, N (%)**												
Personnel	7030	**32.0**	1188	**12.3**	4109	**26.0**	1997	**32.8**	1649	**29.9**	1823	**31.4**
Vaccine	12498	**56.9**	6477	**67.0**	9487	**59.9**	2197	**36.0**	2338	**42.3**	2268	**39.0**
Supplies	175	**0.8**	150	**1.6**	163	**1.0**	83	**1.4**	76	**1.4**	79	**1.4**
Vehicle operation and maintenance	56	**0.3**	56	**0.6**	56	**0.4**	52	**0.9**	32	**0.6**	42	**0.7**
Building operation and maintenance	468	**2.1**	18	**0.2**	243	**1.5**	59	**1.0**	15	**0.3**	37	**0.6**
Community sensitisation and Monitoring	516	**2.3**	671	**6.9**	594	**3.8**	658	**10.8**	658	**11.9**	658	**11.3**
Outreach	-		-		-		112 **1.8**		123 **2.2**		117 **2.0**	
**Total recurrent costs**	**20743**	**94.4**	**8560**	**88.5**	**14652**	**92.6**	**5158**	**84.6**	**4891**	**88.5**	**5024**	**86.5**
**Grand Total**	**21980**		**9676**		**15829**		**6097**		**5525**		**5811**	
**Unit Cost**												
Number of doses administered	4041		2094				711		477			
Cost per dose at 93%	5.4		4.6		5.0		8.6		11.6		10.1	
% proportion of hospital/health centre administered doses	66 %		34 %				60 %				40**%**	
Weighted unit cost per dose	3.6		1.6		5.2		5.1		4.7		9.8	
**% proportion of urban\rural population**	**29 %**						**71%**					
Urban/rural weighted cost per dose					**1.5**						**6.9**	
**Weighted average cost (Urban/rural) per dose**					**8.4**							

Tables 3 and 4 present total and unit cost of diarrhoea management in urban and rural health facilities in more detail. The cost of managing a case of moderate diarrhoea is US$ 2.9 per visit (Table 3) in urban facilities, and US$ 4.2 per visit in rural facilities. Severe diarrhoea management costs US$ 7.6 and US$ 9.4 per bed day in urban and rural health facilities, respectively. Personnel remuneration is the major expenditure, consuming 62% in urban and 39% in rural facilities of the total cost for treating moderate diarrhoea. There is a similar trend for severe diarrhoea with personnel remuneration representing 64% and 42% of total expenditure for urban and rural facilities, respectively.

**Table 3:** **Average outpatient cost diarrhoea treatment per visit, by location and level of service, 2012 US$**

**Cost category**	**Urban**						**Rural**					
	**Hospital**	**%**	**Health Centre**	**%**	**Average**	**%**	**Hospital**	**%**	**Health Centre**	**%**	**Average**	**%**
**Capital Cost, N (%)**												
Buildings	4016	**12.3**	84	**12.2**	2050	**12.3**	61	**4.3**	160	**17.3**	111	**9.4**
Equipment	362	**1.1**	5	**0.7**	184	**1.1**	9	**0.6**	16	**1.7**	12	**1.1**
Vehicles	446	**1.4**	27	**3.9**	237	**1.4**	22	**1.5**	0	**0.0**	11	**0.9**
Training on diarrhoea management	1328	**4.1**	269	**39.0**	799	**4.8**	802	**56.6**	72	**7.8**	437	**37.3**
**Total capital costs**	**6152**	**18.9**	**385**	**55.6**	**3268**	**19.6**	**893**	**63.0**	**248**	**26.8**	**571**	**48.7**
**Recurrent Cost, N (%)**												
Personnel	20371	**62.4**	204	**29.5**	10288	**61.8**	299	**21.1**	622	**67.1**	460	**39.3**
Drugs and Medical supplies	3879	**11.9**	63	**9.1**	1971	**11.8**	58	**19.1**	47	**5.0**	53	**4.5**
Supplies	284	**0.9**	13	**1.8**	148	**0.9**	72	**5.1**	9	**0.9**	40	**3.4**
Vehicle operation and maintenance	413	**1.3**	22	**3.2**	218	**1.3**	23	**1.6**	0	**0.0**	11	**1.0**
Building operation and maintenance	254	**0.8**	5	**0.7**	130	**0.8**	26	**1.8**	0	**0.0**	13	**1.1**
Cleaning and Laundry	1269	**3.9**	1	**0.2**	635	**3.8**	46	**3.2**	2	**0.2**	24	**2.0**
**Total recurrent costs**	**26470**	**81.1**	**308**	**44.4**	**13389**	**80.4**	**523**	**37.0**	**679**	**73.2**	**601**	**51.3**
**OPD Grand Total**	**32 622**		**693**		**16 657**		**1416**		**927**		**1171**	
**Unit Cost**												
Number of annual visit	11 277		247				305		249			
Cost per OPD visit	**2.9**		**2.8**				**4.6**		**3.7**			
**% proportion of Hospital\health centre annual visit**	**98 %**		**2 %**				**55 %**		**45 %**			
**Weighted unit cost per visit**	**2.8**		**0.1**		**2.89**		**2.6**		**1.7**		**4.2**	
**% proportion of urban\rural population**	**29 %**						**71 %**					
**Urban/rural weighted cost per visit**					**0.8**						**3.0**	
**Total weighted average cost per child treated**					**3.8**

**Table 4:** **Average inpatient cost for diarrhoea treatment, by location and level of service, 2012 US$**

**Cost category**	**Urban**					**Rural**				
	**Hospital**	**%**	**Health Centre**	**Average**	**%**	**Hospital**	**%**	**Health Centre**	**Average**	**%**
**Capital Cost, N (%)**										
Buildings	3066	**12.9**	-	3066	**12.9**	277	**10.1**	-	277	**10.1**
Equipment	408	**1.7**	-	408	**1.7**	49	**1.8**	-	49	**1.8**
Vehicles	74	**0.3**	-	74	**0.3**	92	**3.4**	-	92	**3.4**
Training on diarrhoea management	679	**2.9**	-	679	**2.9**	285	**10.4**	-	285	**10.4**
**Total capital costs**	**4227**	**17.8**	-	**4227**	**17.8**	**703**	**25.7**	-	**703**	**25.7**
**Recurrent Cost, N (%)**										
Personnel	15250	**64.3**	-	15250	**64.3**	1141	**41.7**	-	1141	**41.7**
Drugs and Medical supplies	1831	**7.7**	-	1831	**7.7**	163	**5.9**	-	163	**5.9**
Supplies	728	**3.1**	-	728	**3.1**	327	**11.9**	-	327	**11.9**
Vehicle operation and maintenance	30	**0.1**	-	30	**0.1**	98	**3.6**	-	98	**3.6**
Building operation and maintenance	550	**2.3**	-	550	**2.3**	111	**4.1**	-	111	**4.1**
Cleaning and Laundry	1111	**4.7**	-	1111	**4.7**	196	**7.1**	-	196	**7.1**
**Total recurrent costs**	**19500**	**82.2**	-	**19500**	**82.2**	**2036**	**74.3**	-	**2036**	**74.3**
**IPD Grand Total**	**23727**			**23725**		**2739**			**2738**	
**Unit cost**										
in-patient days	3103			3103		291			291	
Cost per in-patient day	**7.6**			**7.6**		**9.4**			**9.4**	
% proportion of urban\rural population	**29 %**					**71 %**				
Urban/rural weighted cost in-patient day			**2.2**					**6.7**		
**Total weighted (urban/rural) average cost per in-patient day**						**8.9**				

### Cost-effectiveness

At baseline, providing only rotavirus immunisation is the least effective of the alternatives, with 1.4 DALYs averted per child, while diarrhoea management alone and vaccine plus diarrhoea treatment in combinations avert 2.0 and 2.5 DALYs per child respectively.

The vaccine alone is also the cheapest of the alternatives with a cost estimate of US$ 59 per child, while the cost of diarrhoea treatment is US$ 112 and the vaccine and treatment in combination is US$ 167 per child. There is no dominance, and the incremental cost-effectiveness ratios (ICERs) are US$ 43 and 112 per DALY averted when moving between the three alternatives (Table 5). Rotavirus vaccine in combination with diarrhoea treatment using ORS and IV fluids is therefore the most cost-effective option compared to the vaccine or diarrhoea treatment alone (Table 5), given that the willingness to pay is at least US$ 112 per DALY.

**Table 5:** **Baseline cost effectiveness results**

**Strategy**	**Cost**	**Incremental cost**	**DALYs Averted**	**Incremental DALYs**	**ICER**
**Discounted**					
No Intervention	0.0	0.0	0.00	0.00	0
Rotavirus Vaccine Alone	59.3	59.3	1.39	1.39	43
Diarrhoea Management	112.2	52.9	1.98	0.59	90
Rotavirus V& Diarrhoea Management	166.7	54.5	2.47	0.49	112

### One-way sensitivity analysis

**Figure 2:**
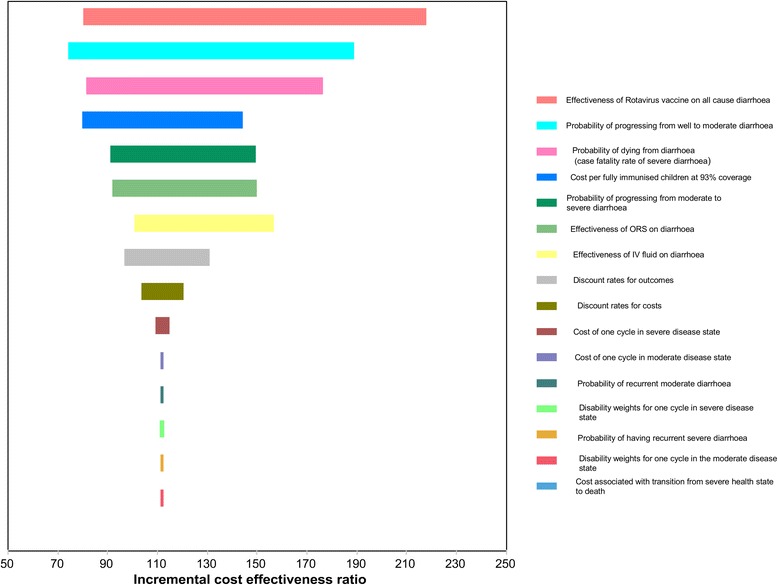
Tornado diagram showing the uncertainty impact of individual parameters on the incremental cost effectiveness ratio. Black dotted line represents the base case ICER. NB: The left hand presents the lower limit ICER values and the right hand upper limit of ICER.

The one-way sensitivity analysis indicates that the vaccine efficacy of diarrhoea is the most influential parameter in the base case analysis (Figure 2). Evaluating the model at the lower limit of the effectiveness of rotavirus vaccine on all cause diarrhoea (0.57), the ICER improved significantly from US$ 112 to US$ 80 per DALY averted, while the upper limit (0.88) predicted a higher ICER of US$ 218 per DALY averted. Other parameters with substantial influence on model results were transition probabilities from well to moderate diarrhoea, diarrhoea case fatality rate, effectiveness of ORS on moderate diarrhoea treatment, transition probabilities from moderate to severe diarrhoea, and the effectiveness of IV fluids on severe diarrhoea treatment and the discount rate for health outcomes.

### Probabilistic sensitivity analysis

**Figure 3:**
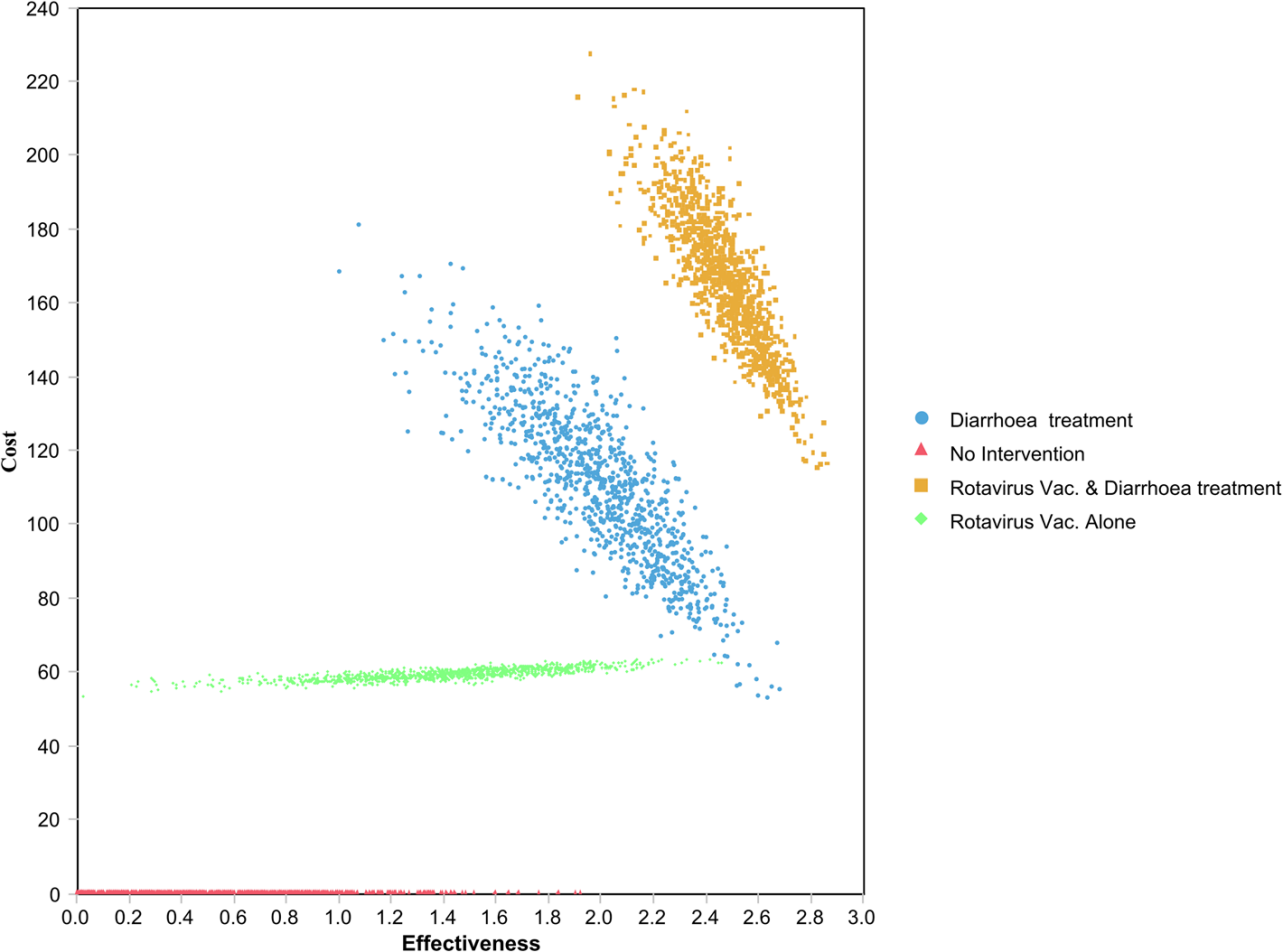
Scatter plot of costs and health outcomes from probabilistic sensitivity analysis.

The probabilistic sensitivity analysis (Figure 3) reveals the combined model uncertainty in cost and effectiveness, and shows that for the rota vaccine alone strategy, uncertainty is largely associated with effectiveness, while uncertainty varies more equally between costs and effectiveness for the diarrhoea treatment alone and the rotavirus vaccine plus diarrhoea treatment.

**Figure 4:**
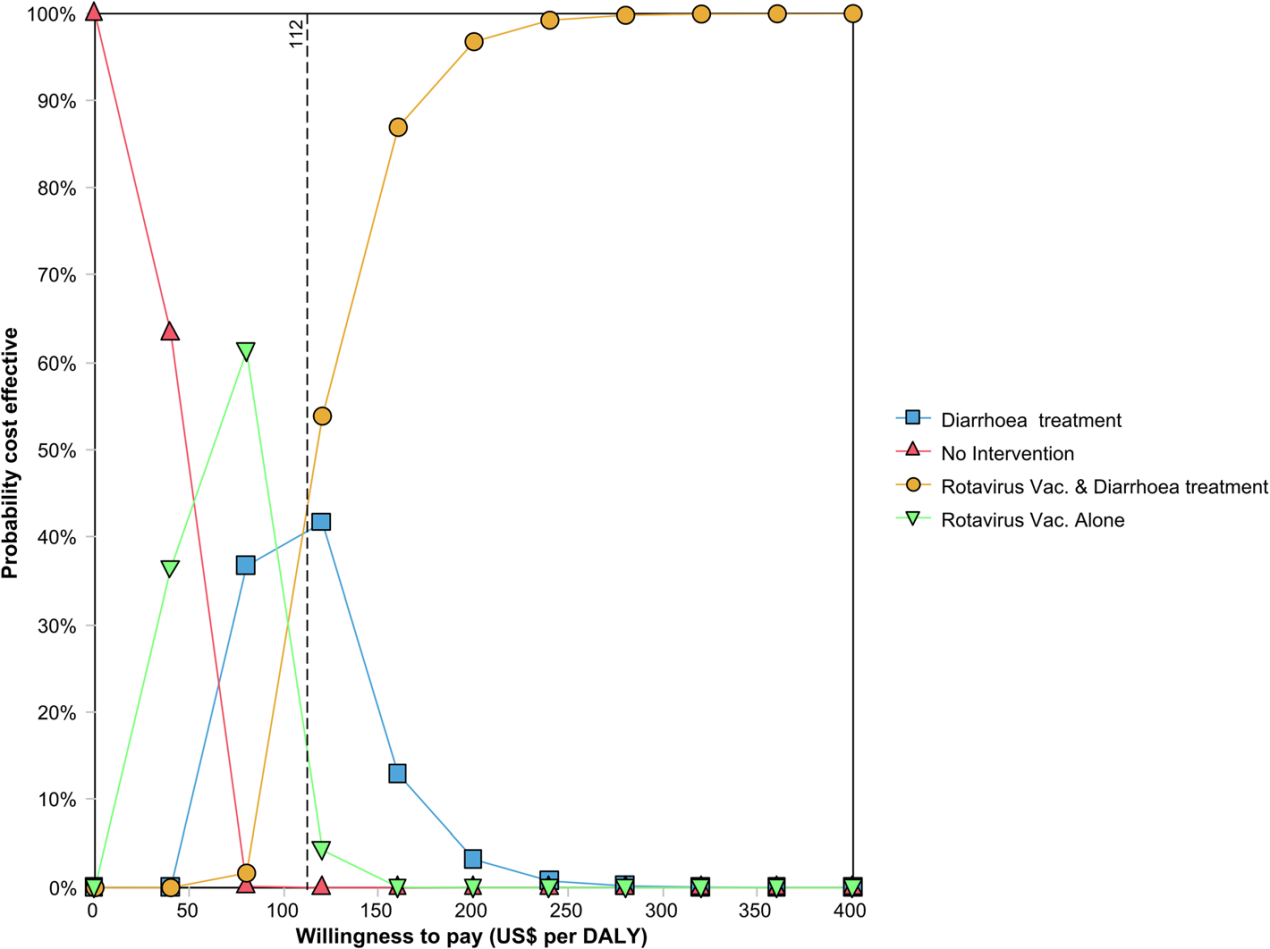
Cost effectiveness acceptability frontier showing the likelihood that any of the diarrhoea management strategy is cost effectiveness for different levels of willingness to pay for health.

The cost effectiveness acceptability frontier (Figure 4) illustrates that willingness to pay to avert a DALY decides which intervention is likely to be most cost-effective. Until willingness to pay to avert a DALY exceeds US$ 40, the null intervention is optimal. For willingness to pay for health between US$ 40 and 80 the vaccine provided alone has the highest probability of being optimal, while in the range US$ 80 to 112 per DALY averted; diarrhoea treatment alone is most likely to be cost-effective. When willingness to pay exceeds US$ 112 per DALY averted the combined strategy of providing both the vaccine and diarrhoea management is likely to be optimal.

Figure 4 also illustrates that there is a large degree of uncertainty surrounding these findings, especially regarding the ranges of willingness to pay for which the monotherapies may be considered optimal. In fact, both these recommendations have less than 60% probability of being cost effective. Uncertainty diminishes only when willingness to pay exceeds about USD 160 per DALY, after which the probability of the combined intervention being cost-effective is higher than 80%. Rotavirus vaccine and diarrhoea treatment combined approaches a 100% probability of being cost effective at a much lower level of willingness-to-pay than the US$609 per capita Tanzanian gross domestic product (GDP) in 2011/2012, suggested by the World Health Organisation as highly cost-effective [40].

## Discussion

This is the first published cost-effectiveness analysis for Tanzania comparing the potential benefit of rotavirus vaccine with diarrhoea management either in combination or if each intervention were implemented separately. We found that rotavirus vaccine provided as a package with diarrhoea treatment is highly cost- effective compared to the implementation of diarrhoea treatment alone or only providing RV1 vaccine. The incremental cost effectiveness ratio remained highly cost effective during sensitivity analysis. One way sensitivity analysis shows that for the most influential parameter i.e. the effectiveness of rotavirus vaccine, the highest ICER is US$ 237 per DALY averted which is lower than Tanzania’s GDP.

The Tanzanian package of essential health interventions and the strategic plan for reduction of maternal and child mortality (2008 to 2015), recommends giving priority to interventions that are cost effective and address the major causes of morbidity and mortality [41,42]. Both policy documents recommend diarrhoea treatment with ORS as a key intervention in diarrhoea control. However, our study shows that diarrhoea treatment alone is likely to be less cost effective than combining it with rotavirus vaccination for reasonable levels of willingness to pay per DALY averted. These findings corroborate the current WHO recommendation on diarrhoea control, emphasising the provision of both prevention and treatment of diarrhoea as a package [10]. We cannot rule out the possibility that local variation in conditions, including epidemiology and capacity for service provision may influence the finding that diarrhoea treatment or vaccine provided alone is less cost effective, but these are unlikely to change the main finding that adding the vaccine is highly cost-effective.

At a unit cost between US$ 5.2 (urban health facilities) to 9.4 in rural facilities per vaccine dose, estimated from subsidised GAVI alliance prices[11] in additional to administrative cost and vaccine wastage from primary cost data. Our study shows that it costs twice as much to deliver the vaccine in the rural facilities as in the urban facilities. This is primarily because there are fewer children in the rural area accessing health care services. Hence there are fewer patients to share the fixed capital costs and the fixed personnel costs of each facility (Figure 2). In other words, both vaccination and diarrhoea treatment are likely to be more cost-effective in urban than in rural areas. Since health services are generally better available and of higher quality in urban areas, this means that scale up of rotavirus vaccination may represent an equity-efficiency trade-off. Prioritizing urban areas will allow more children to be immunized when funds are insufficient for full coverage, but at the same time this will further increase existing disparities. More empirical research is needed to explore the distributive impacts of alternative policies, coupled with deliberation and debate on the normative arguments.

The findings of our study are similar to previous studies on a two-dose monovalent RV1 vaccine in other low-income countries. A study from Malawi reported an ICER value of US$ 75 per DALY averted at vaccine cost of US$ 5.5 per dose [38]. Atherly et al. found a cost of US$ 78 per DALY averted in the WHO AFRO region, at vaccine unit cost of US$7 [37], and a study from India and Kenya also reported that introduction of the monovalent rotavirus vaccine would be highly cost effective [39,43], the unit cost per vaccine in India study was US$7, in the Kenya study the unit cost was between US$ 9.2 and US$ 7.4. However none of these studies directly compared the benefit of combining rotavirus vaccine with diarrhoea management, and all the studies used secondary cost data, either from the WHO-CHOICE project or other vaccination costing studies.

Rotavirus vaccine is expected to provide further societal benefits not captured by our model [44], which only includes the health provider perspective. Even if health services for children in Tanzania are free, the out of pocket expenditure for food, transport, and medicines for diarrhoea are substantial and are estimated to be on average US$ 5.5 per child admission [45]. In addition to these direct costs, indirect costs associated with productivity loss are likely to be highly relevant. Our model therefore probably underestimates the full societal benefits and, consequently, the cost-effectiveness of rotavirus vaccination. The inclusion of RV5 as a comparator might have enhanced the analysis and hence the results, but we chose to exclude the intervention due to lack of Tanzanian cost data.

Cost estimates for diarrhoea management and rolling out the rotavirus vaccine were collected from only one rural and one urban district. Our findings are therefore not necessarily representative for districts that are different in terms of income levels or other characteristics, or for the whole country. Regional estimates could, however, be useful to inform national scale up. The unit costs for diarrhoea treatment were collected in the absence of an immunization programme. After the rotavirus vaccine roll out, the diarrhoea treatment costs might change because of a possible reduction in the number of OPD visits and IPD days. However, we cannot predict that with certainty from our study. We had no apriori evidence suggesting the degree of economies of scale before vaccine introduction. The cost data may be updated after roll-out to reflect possible impact of vaccine on health care expenditure. Our model can easily be adapted using local and updated data to optimize its local relevance.

The effectiveness data used in this work were retrieved from various meta-analyses lacking direct head to head comparisons between competing interventions. The lack of network meta-analysis may impact on the precision of our study results. Ideally network meta-analysis could have been done to further synthesis the evidence, increase precision of the model results and, hence minimise the potential bias of using effectiveness data from several different sources [46]. However these network meta-analysis are only as good as the trials included in them. In the setting in which this study was conducted, these methods are not well developed and it was beyond the scope of this analysis to perform an independent network meta-analysis. Nevertheless, decisions in health care resource allocation have to be made in this context, even in the absence of precision data and more complex analytical and synthesis methods [47]. For further studies, we recommend inclusion of network meta-analysis. It would also be useful if well-established bodies such as the Child Health Epidemiology Reference Group (CHERG) and the Cochrane collaboration consider extending the conventional meta-analysis into network meta-analysis to generate evidence for use in low-income settings.

## Conclusions

A combination of rotavirus immunisation and diarrhoea management for Tanzania is likely to be cost-effective when willingness to pay for health exceeds US$ 112 per DALY. Provisions of RV1 vaccine alone or diarrhoea management alone are both less cost effectiveness alternatives. The roll out of the Rotavirus vaccine as a package with diarrhoea treatment will strengthen the efforts to achieve the child health Millennium Development Goals in Tanzania and should be seen as a high priority intervention for child health improvement.
